# Expectations and needs of patients with a chronic disease toward self-management and eHealth for self-management purposes

**DOI:** 10.1186/s12913-016-1484-5

**Published:** 2016-07-08

**Authors:** Martine W. J. Huygens, Joan Vermeulen, Ilse C. S. Swinkels, Roland D. Friele, Onno C. P. van Schayck, Luc P. de Witte

**Affiliations:** School for Public Health and Primary Care (CAPHRI), Department of Health Services Research, Maastricht University, P.O. Box 616, 6200 MD Maastricht, The Netherlands; NIVEL, Netherlands Institute for Health Services Research, P.O. Box 1568, 3500 BN Utrecht, The Netherlands; Tilburg School of Social and Behavioral Sciences, Tilburg University, Tranzo, P.O. Box 90153, 5000 LE Tilburg, The Netherlands; School for Public Health and Primary Care (CAPHRI), Department of Family Medicine, Maastricht University, P.O. Box 616, 6200 MD Maastricht, The Netherlands; Research Center Technology and Care, Zuyd University of Applied Sciences, P.O. Box 550, 6400 AN Heerlen, The Netherlands; Centre for Care Technology Research, Maastricht, The Netherlands

**Keywords:** Self-care, Telemedicine, Primary health care, Chronic disease, Patients, Health services needs and demand

## Abstract

**Background:**

Self-management is considered as an essential component of chronic care by primary care professionals. eHealth is expected to play an important role in supporting patients in their self-management. For effective implementation of eHealth it is important to investigate patients’ expectations and needs regarding self-management and eHealth. The objectives of this study are to investigate expectations and needs of people with a chronic condition regarding self-management and eHealth for self-management purposes, their willingness to use eHealth, and possible differences between patient groups regarding these topics.

**Methods:**

Five focus groups with people with diabetes (*n* = 14), COPD (*n* = 9), and a cardiovascular condition (*n* = 7) were conducted in this qualitative research. Separate focus groups were organized based on patients’ chronic condition. The following themes were discussed: 1) the impact of the chronic disease on patients’ daily life; 2) their opinions and needs regarding self-management; and 3) their expectations and needs regarding, and willingness to use, eHealth for self-management purposes. A conventional content analysis approach was used for coding.

**Results:**

Patient groups seem to differ in expectations and needs regarding self-management and eHealth for self-management purposes. People with diabetes reported most needs and benefits regarding self-management and were most willing to use eHealth, followed by the COPD group. People with a cardiovascular condition mentioned having fewer needs for self-management support, because their disease had little impact on their life. In all patient groups it was reported that the patient, not the care professional, should choose whether or not to use eHealth. Moreover, participants reported that eHealth should not replace, but complement personal care. Many participants reported expecting feelings of anxiety by doing measurement themselves and uncertainty about follow-up of deviant data of measurements. In addition, many participants worried about the implementation of eHealth being a consequence of budget cuts in care.

**Conclusion:**

This study suggests that aspects of eHealth, and the way in which it should be implemented, should be tailored to the patient. Patients’ expected benefits of using eHealth to support self-management and their perceived controllability over their disease seem to play an important role in patients’ willingness to use eHealth for self-management purposes.

**Electronic supplementary material:**

The online version of this article (doi:10.1186/s12913-016-1484-5) contains supplementary material, which is available to authorized users.

## Background

Self-management is considered as an essential component of chronic care by primary care professionals. People with a chronic disease, such as diabetes, chronic obstructive pulmonary disease (COPD) or a cardiovascular condition, have to make day-to-day decisions to manage their own disease. Self-management requires an active role of the patient in managing one’s symptoms, treatment, physical and psychosocial consequences and lifestyle changes [1]. For example, patients make decisions about medication intake, participation in sports and daily activities and about other lifestyle behaviour, such as adhering to a special diet or giving up smoking. In addition, they have to deal with emotions such as anger, frustration, and depression, which are often inherent to living with a chronic disease. Patients who engage in optimal self-management behaviour improve their quality of life and health outcomes [1–3].

Performing optimal self-management behaviour is difficult and demands a substantial effort from the patient. Previous research has shown that patients with a chronic condition perceive many barriers to engaging in active self-management [4], such as controlling weight, exercising regularly, fatigue, pain, depression, lack of family support and poor communication with physicians.

eHealth technologies that patients can use at home are expected to play an important role in supporting patients in their self-management. eHealth is a broad term which includes a diverse range of technical innovations in healthcare. Eysenbach defined it as ‘an emerging field in the intersection of medical informatics, public health and business, referring to health services and information delivered or enhanced through the internet and related technologies…’ [5]. However, this should be considered with caution, because he also argues that it is very difficult to set up a clear definition of eHealth because of its dynamic environment; ‘stamping a definition on something like e-health is somewhat like stamping a definition on ‘the Internet’: It is defined how it is used - the definition cannot be pinned down, as it is a dynamic environment, constantly moving’ [5].

A diverse range of eHealth technologies are aimed to support patients in their self-management. For example, e-coaching and activity monitoring applications can support and inform patients regarding diet, exercise and weight control by providing insight into self-monitored data, tailored information and feedback, and encouragement [6]. In addition, e-coaching applications can assist patients with depression and anxiety [7] and electronic communication enables patients to communicate effectively with their health-care professionals [8, 9]. Moreover, home telemonitoring applications for people with a chronic condition can produce accurate and reliable data, empower patients, influence their attitudes and behaviours and potentially improve their medical conditions [10]. Although expectations of the use of eHealth are positive, Peeters et al. [11] conclude that up until now there is not enough convincing evidence that care technologies have a positive effect on patient self-management.

In addition, often eHealth is not being adopted successfully in daily care routines [12]. One of the reasons for this is the non-use of eHealth by patients. Reported arguments for non-use and withdrawal by patients are the lack of perceived additional benefits of eHealth, the view that the regular health care is sufficient [13, 14], technological difficulties with the equipment and the association of eHealth with a high degree of dependency and ill health [14].

Another problem is that eHealth is often not tailored to individual patients [12]. Before implementing new eHealth technologies it is important to take into account how individuals currently manage their disease and the ways they adapt to their chronic condition. Van Houtum et al. [15] found in a quantitative study that self-management tasks are partly disease-specific and partly generic. People with diabetes or a neurological disease perceive more daily self-management tasks compared with people with another chronic condition such as COPD or a cardiovascular disease. Understanding the fit between everyday routines and eHealth is an essential part for a successful uptake and use [12].

Before eHealth can be effectively implemented and used, it is important to involve patients in investigating their needs and requirements regarding the use of eHealth. User-centred design (UCD) is a frequently used method to involve patients during the design and development of eHealth [16, 17]. Most studies have used UCD to improve the functionality and usability of an eHealth technology. However, less effort has been put into the step before design and eHealth development. In what aspects of self-management do people with a chronic condition need additional support, and if they need support, are they actually willing to use eHealth?

The aims of the current study are to investigate the expectations, opinions and needs of people with a chronic disease regarding aspects of self-management in which they prefer additional support, and toward eHealth for self-management purposes. In addition, the aim is to investigate patients’ willingness to use such kinds of eHealth technologies. To investigate possible differences between patient groups, patients with 1) COPD, 2) diabetes and 3) a cardiovascular condition were included. These patient groups are included because they belong to the main chronic disease types worldwide [18], care standards for these diseases are developed in the Netherlands (see Additional file 1) and these groups might benefit from eHealth for self-management purposes.

## Methods

### Recruitment and design

People with diabetes, COPD, and a cardiovascular condition were invited to participate in a focus group. They were recruited in four primary care centres in the Netherlands by their care professional. Inclusion criteria were: patients had to be aged over 18 and diagnosed with COPD, diabetes or a cardiovascular disease. Exclusion criteria were: severe psychiatric illness or cognitive impairment, or an insufficient mastery of the Dutch language leading to not understanding the information about the study. People who were interested received an information letter, informed consent form and a questionnaire. The questionnaire was used to collect some background information of the participants and consisted of three short questions: ‘What kind of chronic diseases do you have? ’; ‘Have you already used care technology (for example searching for information about your disease on the Internet, using an online coach or using a self-monitoring system)? ’; and ‘How easy or difficult do you find using the Internet? ’. Focus groups were planned when at least six participants with the same chronic condition agreed to participate. The goal was to organize two focus group interviews for each chronic condition. A researcher (MH) or a care professional contacted the participants to schedule a date and time for the focus group. After the recruitment of people with diabetes in one primary care centre, only four individuals agreed to participate. Therefore, three individuals with diabetes who were under treatment in another primary care practice were invited by the researcher to participate in that group. All participants provided written informed consent and filled out the questionnaire. The study was approved by the Medical Ethical Committee Atrium Orbis Zuyd (METC number: 14-N-86).

Five focus groups were conducted between October 2014 and May 2015. Each focus group took place in the primary care centres where the participants were recruited.

### Procedure

All focus groups were moderated by MH and an assistant moderator (JV or a research assistant). After an introduction about the goal and procedure, the following themes were discussed: 1) the impact of the chronic disease on patients’ daily life; 2) their opinions and needs regarding self-management; and 3) their expectations and needs regarding, and willingness to use, eHealth for self-management purposes. With regard to this last theme, three different types of eHealth applications were discussed: 1) self-monitoring tools in which patients can monitor their own health data and share these with their health-care professionals via the Internet; 2) online coaches in which patients can get advice about their disease or lifestyle; and 3) online communication applications, such as online video consultation or email-consultation. By discussing this theme, participants were first asked whether they had ever used technologies or the Internet for health purposes and what they knew about the possibilities of other eHealth technologies. The different eHealth technologies that participants came up with were discussed. The moderator added other possibilities of eHealth technologies to make sure that the three different types of eHealth technologies were similarly discussed in every focus group.

The moderator’s role was to briefly introduce the themes, to encourage participants to share their thoughts and to ask follow-up questions for clarifying opinions.

Each focus group lasted approximately two hours, was audiotape recorded, and the assistant moderator collected written field notes.

### Data analysis

All focus groups discussions were transcribed verbatim by a research assistant. Afterwards, MH checked the transcripts against the audio recordings. First, two researchers (MH and JV) independently analysed one transcript of a diabetes, COPD and cardiovascular group. Because of the exploratory nature of the focus group set-up, a conventional content analysis approach [19] was used for coding. The researchers checked for consensus of the different codes in the three transcripts. MH used this coding scheme for the remaining two transcripts. New codes were added when necessary. Then, MH and JV clustered the codes and agreed on the main- and sub-themes of the coding scheme. The transcripts were coded using NVivo version 9.

## Results

### Participants

A total of 30 participants with a mean age of 68 years (range 50–83) took part in the focus groups. Of these, 73 % were male. Two focus groups were conducted with people with diabetes (*n* = 7 and *n* = 7), two with people with COPD (*n* = 4 and *n* = 5) and one with people with a cardiovascular condition (*n* = 7). Four individuals with COPD did not show up (one with a given reason). Table 1 presents an overview of the characteristics of the participants.

**Table 1:** Characteristics of the study sample

Characteristics		Mean (range) or n (%)
Age in years	People with diabetes	67.1 (51–79)
	People with COPD	60.3 (50–81)
	People with a cardiovascular disease or CVRM	72.1 (55–83)
Gender: number and percentage of males	People with diabetes	11 (78.6 %)
	People with COPD	6 (66.7 %)
	People with a cardiovascular disease or CVRM	5 (71.4 %)
Internet usage	People with diabetes	
	*Did not use the Internet*	-
	*Very difficult or difficult*	2 (14.3 %)
	*Neutral*	-
	*Easy or very easy*	12 (85.7 %)
	People with COPD	
	*Did not use the Internet*	1 (11.1 %)
	*Very difficult or difficult*	-
	*Neutral*	5 (55.6 %)
	*Easy or very easy*	3 (33.3 %)
	People with a cardiovascular disease or CVRM	
	*Did not use the Internet*	1 (14.3 %)
	*Very difficult or difficult*	1 (14.3 %)
	*Neutral*	-
	*Easy or very easy*	5 (71.4 %)

*COPD* chronic obstructive pulmonary disease*, CVRM* cardiovascular risk management

Participants with COPD mentioned that they visited the practice nurse or general practitioner (GP) one to two times a year. Most of them had a mild to moderate severity of COPD. One participant suffered from COPD GOLD (Global Initiative on Obstructive Lung Disease) stage IV, which means a very severe COPD. This participant visited the pulmonologist four times a year (P4). Four participants reported that they had been under treatment by a physiotherapist (P1, P2, P6 and P8). All participants reported that they used oral medication one or two times a day in forms of pills and/or inhalers. One participant had once had an email-consultation (P6). The others had never used an eHealth technology.

Participants with diabetes reported that they visited the practice nurse or GP two to four times a year. Five participants injected insulin (P2, P3, P4, P8 and P9), eight participants took only oral drugs, and one participant did not use medication (P1). Four participants had had email contact with their care professional (P3, P4, P7, and P8). One participant used a diabetes manager application to get overviews of blood glucose values, which could be sent to his practice nurse, and a medication reminder application (P3). One participant had used an online diabetes coach whereby he could insert his blood glucose values to get advice (P8). Two participants used a food diary application (P13 and P14).

Participants with a cardiovascular condition reported that they visited the practice nurse or GP one to four times a year. Most of them reported having an annual check with the GP. Three participants mentioned that they visited the cardiologist once a year (P1, P3 and P6). Four participants reported having high blood pressure and a high level of cholesterol (P1, P2, P3 and P7), one participant also had a heart rhythm disorder and had received cardioversion (P3). Three participants had a stent placed and/or had received angioplasty (P4, P5 and P6), of whom one had suffered a heart attack (P5). All participants used oral medication. One participant had had email contact with a care professional (P3). None of the others had used an eHealth technology.

### Themes

Based on the analyses, four main themes are identified: 1) opinions and needs regarding self-management support; 2) general requirements regarding eHealth usage; 3) general requirements regarding the implementation of eHealth; and 4) costs and budget cuts in care. Table 2 presents an overview of all main- and sub-themes.

**Table 2:** An overview of the identified main- and sub-themes with the associated topics

Main theme	Sub-themes	Topics
Opinions and needs regarding self-management support	Information	- Need for information (treatment, complications, medication and life style) - Sources of information
	Drug management support	- Need for drug management support - Determining whether medication is necessary - Forgetting to take medication
	Symptom management support	- Need for symptom management support - Need for self-monitoring support - Insight into health status
	Support for management of psychological consequences	- Anxiety regarding further complications - Disease acceptance - Anxiety regarding self-monitoring
	Lifestyle	- Sports - Nutrition and diet - Smoking - Motivation for lifestyle changes
	Social support	- Using support of family and relatives
	Communication	- Current communication with care professionals - Opinions regarding online communication - Need for (online) communication
General requirements regarding eHealth usage	- Usability - Reliability of technology - Trust in the Internet - Unable to use the Internet	
General requirements regarding the implementation of eHealth	- eHealth should support care - Using eHealth should be the choice of the patient - Clear instruction should be given	
Costs and budget cuts in care	- Current costs in care - Costs of eHealth - Budget cuts in care	

#### Opinions, expectations and needs regarding self-management support

##### Information

In general, the majority of participants mentioned that at the moment they had no need for more information about their disease. Most information was gathered from care professionals, and many participants reported that they gathered information on the Internet and in brochures. Many participants responded that there is sufficient information available, especially on the Internet; it is down to the patient to search for it and to decide what to do with the information. Most people agreed that the patient, not the care professional, is most responsible for their health.

*‘We* [practice nurse and patient] *discuss together what seems to be the best, and I feel comfortable with that. But of course I look on the Internet, and of course I read brochures and books, and of course I listen to what they* [care professionals] *say. Nevertheless, I try to use my own sense and think: well, it is my body. So that’s the combination I’m looking for. I feel comfortable with that.’* Diabetes, P6

*‘There is no lack of information; you can gather information everywhere, from the Internet, for example. But what we need is a little bit of discipline. Yes, I don’t have it myself, but I know I need it.’* Diabetes, P11

A few participants with diabetes or a cardiovascular disease mentioned that they wished they had been better informed about the risks and consequences of their disease when it was diagnosed, so they could have been more aware of the consequences of their lifestyle at that time, and thus further complaints could have been prevented.

Several participants in all patient groups mentioned getting anxious from the information they find online. Particularly when reading information about complications that could occur in a later stage of their disease.

##### Drug management

Differences are found in experiences and needs regarding drug management between patient groups.

The majority of participants mentioned that taking medication is a daily routine now, although they reported that they frequently forgot to take their medication during the first period of their disease. However, participants with mild complaints of COPD reported that they still frequently forget to take their medication, because they do not feel that it has any effect on their condition. Because of this, some just decided to stop using the medication. Others have discussed it with their carer.

One participant with diabetes used a medication management application on his mobile phone, which reminded him to take medication. He mentioned that due to this application, having diabetes was no issue for him. Several others in all patient groups liked the idea of using a medication management application. Some already used a pillbox to manage their medication.

Drug management played an important role in the life of people with diabetes. Most participants who inject insulin measured their blood glucose level daily. People with a stable blood glucose level for a long period of time, or people who used oral medication, only monitored their blood glucose level at moments when feeling not well. Participants who measured their blood glucose level discussed these values during regular consultations with the GP or practice nurse. Participants who frequently measured their blood glucose level often consulted the practice nurse in between consultations by email or phone to check whether the level of insulin intake needed changing. These people responded that they had a need for an application that automatically sends their blood glucose data to their practice nurse, so he or she could respond to it. In this way, participants mentioned, insulin intake could be adapted sooner to their actual health status. Customized or individual care was a frequently mentioned benefit of sending self-measured data and receiving feedback on this.

*‘If you can monitor automatically, you get customized care more quickly. Currently, you’re going to the care practice four times a year, and in the period in between you stay at the same value* [of insulin], *while you maybe should have changed it in the meantime, but you didn’t know that.’* Diabetes, P8

One participant with diabetes had used an online diabetes coach whereby he could insert his blood glucose values to get advice. Although he mentioned that this could be really useful, especially for people who are just starting to use insulin injections, or when blood glucose values highly fluctuate, he did not use it anymore because it was not working properly.

The general view of people with a cardiovascular condition about medication intake was that they just did it, because it was advised by their care professional.

##### Symptom management

Expectations and needs regarding symptom management differed between patient groups.

Participants with COPD had mixed opinions regarding monitoring lung function or saturation at home and getting more insight into their health status. Some mentioned that they are interested in using self-monitoring tools at home to check how it is going and to investigate declines to prevent further complaints. These participants liked the idea that care professionals also have insight into these data, so they can advise them whether they should go to a consultation. In contrast, others mentioned that they did not perceive any benefits in monitoring lung data at home. They commented that they could feel if there was something wrong and at such moments they could immediately make an appointment with their GP or practice nurse.

*‘You can probably detect your complaints a little earlier and prevent getting such pulmonary constrictions. I think prevention is an important advantage.’* COPD, P6

*‘At a certain moment you know your own body so well, you know your lungs, you know your breathing, you know your sputum, so you just know at a certain moment that it’s going in the wrong direction. Then, you just call the care professional, and he or she takes immediate action, so why should I do all of this at home?’* COPD, P4

Most people with diabetes who inject insulin monitored their blood glucose level regularly, made overviews of these data and sent it by email to their practice nurse. One participant used a diabetes manager application that tracked his blood glucose values in logbooks and showed him overviews of these data, which could be sent to his practice nurse. He found this application very useful. Many participants suggested that the option of automatically sending data of every measurement to the care practice would be useful because the care professional then has up-to-date data and can respond to it when there are deviations in that data. Most participants with diabetes mentioned the benefits of tracking and sending blood glucose values to their practice nurse. The most frequently reported benefits were: preventing further complaints, getting advice on whether a consultation was needed, and doing more at home instead of going to consultations in the care practice.

‘*By taking measurements every day and sending them to the care practice, for Mister X* [a person who has a stable blood glucose level for years] *no alarm bells will ring, but for me they probably will, if it* [the blood glucose level] *is low and then high again, and when I’m dizzy, then the practice nurse could say, hey, that’s not going well. She could notice that at her computer screen in the morning, so she doesn’t have to read all those emails.’* Diabetes, P13

The majority of participants with a cardiovascular condition reported that the disease had little impact on their daily life and that they had few complaints. Most of them commented that they perceived no benefits in measuring symptoms at home. The regular health checks at the practice nurse or GP were sufficient for them. One participant mentioned that he did not have the feeling of being a patient. By measuring blood data at home he would be more aware of his condition, which he perceived as a negative feeling.

##### Management of psychological consequences

Mixed opinions are found regarding the management of psychological consequences in all patient groups. Some participants with COPD and diabetes mentioned having had a panic attack due to severe health complaints of their chronic disease (e.g. hypoglycaemia and exacerbation attacks). In particular, attacks during the first period of their disease were accompanied with high feelings of anxiety, because they did not know what to do.

Several participants with COPD and diabetes reported being anxious about further complications of their disease. Participants with COPD in particular mentioned that they had experienced a decline in energy levels. Some participants with COPD or diabetes mentioned knowing from relatives, acquaintances or the Internet what could happen in the next stage of their disease (e.g. insulin injections, supplemental oxygen intake or death) and were wondering how their condition would further develop in the upcoming years.

*‘You also become mentally tired of it – knowing that you have a disease, that disease will never disappear – but where will this end? This disease stays on your mind. More than you actually want.’* COPD, P6

In addition, a few participants with COPD and diabetes blamed themselves for having the chronic disease because of an unhealthy lifestyle. Participants with a cardiovascular condition were least concerned about their chronic condition.

Talking about self-monitoring applications, participants with COPD and a cardiovascular condition reported that they would expect to feel increased feelings of anxiety due to monitoring health data at home. Anxiousness because of doing the measurements themselves, and not knowing what to do with deviant data were frequently mentioned as expected negative consequences of self-monitoring at home. In addition, many participants in all patient groups reported the disadvantage of frequently being reminded of having a chronic condition.

*‘It will also cause disturbance, when you have to do all of this at home* [monitoring]*. Imagine doing that in the evening at 10 o’clock and then you feeling unwell, what should you do? Then you have to wait the entire night, because there will be nobody here* [in the primary care practice]. *I really don’t like that idea.’* COPD, P3

‘*The disadvantage is that I’m feeling more like a patient* [because of frequently monitoring]*: man suffers most from the suffering he fears.’* Cardiovascular condition, P7

##### Lifestyle

The types of lifestyle behaviour that were most frequently discussed differed between patient groups. Furthermore, the role that a healthy lifestyle played in participants’ lives differed among patient groups.

Exercising and giving up smoking were frequently discussed lifestyle behaviours among participants with COPD. Although most of them were aware that this is important for their health, some had difficulties in keeping this up.

Nutrition and diet were most discussed by participants with diabetes. They mentioned that nutrition immediately affected their blood glucose level and thus their health status. A few of them were treated by a dietician. Two participants used an online food diary application which effectively helped them to choose what to eat.

Participants with a cardiovascular condition mentioned the importance of a healthy lifestyle, but this played a less important role in their daily life compared with participants with diabetes and COPD.

Many participants in all patient groups reported that enough sources are available to raise awareness of how to live a healthy life. Advice is gathered from care professionals, brochures or the Internet. Many of them had no interest in using an online coach to motivate and stimulate them to change their lifestyle, such as an online coach to help give up smoking or a food diary application. Intrinsic motivation to give up smoking, lose weight or do more physical exercises was seen as most important.

Several participants in all patient groups reported that when they were diagnosed they had been warned that they should change their lifestyle to prevent further complications. However, at that time they were less aware of the risks and consequences, and therefore they did not change. They mentioned that it is really important that care professionals create awareness of the risks and consequences of the chronic disease.

*‘She* [the practice nurse] *mentioned that it is bad for the organs if you eat sugar and that sort of things, and that’s it…. And then you just continue your life, and then you get a pill…. Then the pressure is not that high. But eventually you have to use insulin, and yes, that could be prevented, I think, if that awareness happened earlier.’* Diabetes, P8

##### Social support

Using the help and knowledge of relatives was frequently reported by participants in all patient groups. Some participants had family members with a medical background or the same chronic disease who advised them. Others had family members who reminded them to take their medication, or searched for them on the Internet for information about their chronic disease. In addition, several persons mentioned that they contacted their children or grandchildren when they needed help with technical problems with computers or the Internet.

On the other hand, a few participants with COPD and diabetes mentioned that they did not want to show others that they have a chronic disease, and were afraid of scaring people by using a self-monitoring application. In addition, some people with COPD had difficulties in explaining to family members that they have limited energy to do activities due to their COPD. Many participants in all patient groups expected that support from relatives and family members would become more important because of cuts in care.

##### Communication

Many participants in all patient groups mentioned that they have found the regular number of practice visits convenient. In particular, communicating with the practice nurse was perceived as agreeable. In between the regular consultations, there was always the possibility of calling or emailing the practice nurse or GP, and if necessary to visit the care practice within short notice.

Only a few people had experiences with online communication with care professionals. Participants with diabetes most frequently reported that they had email contact with the practice nurse about their self-measured blood glucose level. They had found this convenient.

Many participants reported that they expected having an e-consultation (asking a question via email or an online program) would be very impersonal and cold, because of the lack of eye contact and interaction. They also questioned within what time span they would receive answers. In addition, they mentioned that it would be difficult to describe their complaints and fears by typing. An online video consultation using a webcam was expected to be more convenient compared with an e-consultation, because of the direct contact with the care professional. However, many participants mentioned that when they urgently need a care professional, they would prefer visiting him or her in real life. Several participants added that the ‘older generation’ is just not used to communicating over the Internet.

*‘I think I find it more pleasant to have eye contact with the care professional, so when I ask a question I can see their face, and what he or she thinks of it…. And I also think, will I get an answer immediately or in two days?’* COPD, P4

Reported needs for (online) communication were that communication should be direct, understandable, tailored to the patient, and conducted by a human, not by a preprogrammed application.

#### General requirements regarding eHealth usage

Many participants in all patient groups responded that eHealth should be easy to use, and should require as few actions as possible, in particular for older people who are not familiar with the Internet or modern technologies. In addition, self-monitoring tools should be easy to carry. Moreover, eHealth should be reliable and function properly. The unreliability of home blood pressure meters and non-functioning websites were mentioned as bad examples.

A well-discussed topic in all patient groups was trust in the Internet. Most participants reported having no problems with sending and sharing data over the Internet. Many participants talked about the advantages of a national electronic health record (which has not yet been introduced in the Netherlands). However, in every patient group a few people did not trust the Internet because of previous experiences or rumours in the media about data leakage. In addition, some worried that non-medical people would get access to their health data, such as insurance companies and managers.

*‘Yes I’m using the Internet and so on, but I don’t use it for everything that is personal… I don’t trust it. Maybe I’m old-fashioned, but I don’t trust it. Sometimes I read in the newspaper that DigiD* [digital identity for Dutch governmental websites] *is already unsafe.’* Diabetes, P12

*‘When I have to go to the night care clinic, or when I have an accident, it’s totally fine that they* [care professionals] *have access to my medical data. But what I don’t want is that my health and safety officer gets insight to see my medical data, and tells everything to my manager’* COPD, P4

Moreover, a few participants mentioned that they were not able to use computers or the Internet, or are not interested in using it. Others referred to friends or family members of a similar age who did not have the right skills or interests.

#### General requirements regarding the implementation of eHealth

The general view about the implementation of eHealth was that it should not be compulsory: the patient should be allowed to choose whether or not to use it. Some participants mentioned being afraid that patients will be forced to use care over the Internet, and compared it with online banking and the Dutch tax authority’s website (in the Netherlands an extra amount has to be paid for not banking online, and the standard procedure for arranging tax returns is via the Internet). In addition, participants reported that patients who use eHealth should also be given the opportunity to receive regular, and in particular personal care; eHealth should support care, but not replace personal care.

*‘It should be nuanced and individualized: those who are able to do it, and like it, yes okay, but if someone is not yet ready for it, or doesn’t like it, give them the opportunity to fill it in in a different way.”* Cardiovascular condition, P1

Several participants mentioned that because of the rapid development of modern technology, the implementation of eHealth cannot be stopped. Some were concerned about this, while others liked the idea of implementing innovations in health care.

*‘We are moving in that direction anyway, whether we like it or not… 10 or 15 years ago we did not even know what a bank card was, and now it’s very common, now we pay by card at the cash register. And that’s also how it will go with care technology, I’m convinced about that. And if we don’t follow that trend we’ve got it wrong.’* Diabetes, P7

If eHealth is to be introduced and offered, participants preferred that the care professional would clearly show how it can be used. Furthermore, clear instructions should be given via digital or written manuals or via YouTube videos.

#### Costs and budget cuts in care

Costs and budget cuts in care were frequently mentioned topics in all focus groups. Many participants complained that nowadays health-care costs are higher compared with several years ago. They expected that costs would continue to rise in the coming years because of budget cuts in care. Several participants mentioned that they expected that costs would increase because of the implementation of eHealth. Several participants expected that health insurance companies would decide what kind of eHealth patients should use, like they do now in the choice of medication because of deals between pharmaceutical companies and insurance companies. Others reported that they had heard that eHealth has been developed because of budget cuts in care and because of its cost-effectiveness.

*‘In whose interest is it to develop these technologies and innovative things anyway? It’s the result of less money and fewer doctors.’* Cardiovascular condition, P1

## Discussion

### Principal results

This qualitative research showed indications of differences between patient groups in their expectations and needs regarding self-management and eHealth for self-management purposes. In general, people with diabetes reported the most needs and benefits regarding self-management aspects and were most willing to use eHealth, followed by the COPD group. In contrast, people with a cardiovascular condition mentioned having fewer needs for self-management support because their chronic condition had little impact on their daily life. Each patient group reported similar general requirements for eHealth. In addition, it was reported that the patient, not the care professional, should choose whether or not to use eHealth. Moreover, participants reported that eHealth should support care and not replace personal care.

All self-management constructs identified in Barlow et al. [1] were identified in the current study as sub-themes regarding self-management. In general, more opinions were investigated regarding information, drug management, symptom management, communication and lifestyle than regarding management of psychological consequences and social support. Participants might have been less open to share their personal experiences and needs regarding disease acceptance and emotional consequences inherent to living with a chronic condition. In addition, the main focus of the Dutch approach to self-management, as in many other European countries, is on medical and behavioural management, and less on helping patients in dealing with emotional consequences [20]. Participants were therefore maybe less focused on these topics when talking about their experiences of dealing with their chronic disease.

The differences between people with COPD, diabetes and a cardiovascular condition in expectations and needs regarding self-management and eHealth might be related to differences in treatment, symptoms and degree of manageability among the disease types. Many people with diabetes were already familiar with self-monitoring applications for measuring blood glucose level. In addition, people with diabetes reported that nutrition, weight loss (to achieve a normal weight) and medication directly influenced their health. Therefore, they might perceive that their disease is more controllable by their own behaviour, which could influence their interests in eHealth for self-management purposes. In contrast, people with a cardiovascular condition mentioned having few complaints and reported that their disease had little impact on their daily life. Therefore, many people commented that they perceived no need for eHealth for self-management purposes. People with COPD had mixed opinions regarding self-management support. Although psychological consequences of the chronic illness were less discussed, people with COPD more frequently mentioned that their health status had declined during the past years, and were wondering how their disease would further develop in the upcoming years. This might indicate the feeling of having less control over their disease, which could limit the added value of using eHealth for self-management support.

Patients’ expected benefits of using eHealth to support self-management might be the most important predictor of patients’ willingness to use such kind of eHealth. The factor ‘perceived usefulness’ is included in widely used technology acceptance models [21, 22]. Based on this study, it can be suggested that the perceived benefits should outweigh the negative consequence of frequently having to take action to deal with the disease, which reminds patients about having a chronic condition. In addition, it seems that when patients already function in (social) systems that provide them sufficient knowledge and support, they will be less interested to use an eHealth technology for these purposes. In this research this was meanly found for social support and lifestyle. It can be argued that, in the case the (social) system is insufficient or radically changing, patients’ perceived benefits and likewise their interests in eHealth might improve.

Moreover, it is indicated that different patient groups have different needs regarding additional self-management support. Therefore, patients’ perceived benefits could increase when eHealth is tailored to the patient group. In addition, previous research has found that people with COPD who had no experience with eHealth had no clear ideas about the advantages [23]. Therefore, it should be important to clearly inform patients about the possible benefits of using eHealth when introducing it.

Moreover, patients’ interest in self-management support might be dependent on the controllability patients believe to have over their disease. The concept of health controllability, better known as health locus of control, is found to be a factor that influences health-related behaviour [24, 25]. Some studies suggest that patients with a high internal locus of control may be more attracted to self-management interventions [26, 27]. Future research should be performed to investigate the relation between patients’ believed controllability over their disease and their willingness to use self-management support technologies.  Many participants reported that they expected feelings of anxiety by taking measurements themselves, or because of not knowing what to do with deviant data of those measurements. Therefore, it is recommended that health-care professionals should clearly inform and show patients how to use eHealth and inform them how to interpret the results. In addition, many participants associated the implementation of eHealth with budget cuts in care, and some expected that its use would be imposed by health insurance companies. Giving patients the choice of whether or not to use it, and clearly informing them about the reasons for its implementation will be important. In addition, independent sources, such as patient associations and health-care organizations, could play an important role in informing and stimulating patients to use eHealth. However, even when these recommendations are taken into consideration, it is important to keep in mind that not all patients are willing to use eHealth. Several participants mentioned that they did not want to use the Internet for health purposes, that they are not able to use eHealth, or that they are just not interested in using it.

### Strengths and limitation

In previous studies, the expectations and needs of patients regarding the use of eHealth to support self-management have been frequently investigated for specific applications. The strength of this study is its focus on self-management and eHealth in general; people with a chronic condition could express their own ideas, needs and interests. In addition, by using a qualitative methodology participants were not forced to value their needs from fixed options, but they could respond and explain their needs using their own words and ideas [28]. Moreover, a strength of this study is that people with the same chronic condition participated in one focus group, resulting in in-depth discussions in which disease-specific needs could be investigated.

A limitation of this study is that only one focus group with people with a cardiovascular condition was conducted. Some care professionals were not able to recruit enough participants with this condition, or preferred to organize a focus group with people with diabetes or COPD. Another limitation is the high percentage of males who participated in this study. One reason might be that the word ‘care technology’ had a deterrent effect on female patients that were invited. In addition, lower reported Internet skills were found in the COPD group compared with the other two groups. Although most of these participants were still familiar with the Internet, this could have influenced the results concerning willingness to use eHealth, since it is found that the degree of computer and Internet skills has an influence on technology acceptance [29].

## Conclusion

Differences are found in expectations and needs between different patient groups regarding self-management and eHealth for self-management purposes, suggesting that eHealth and its implementation should be tailored to the patient group. Patients’ expected benefits of eHealth and their perceived controllability over their disease seem to be important in patients’ willingness to use eHealth for self-management purposes. Informing patients clearly in a well-considered way about the possibilities, usage and reasons for implementation are important for stimulating the uptake of eHealth in primary care. However, when offering eHealth to patients it should be taken into account that not every patient is willing to use it.

## Abbreviations

COPD, chronic obstructive pulmonary disease; CVRM, cardiovascular risk management; GP, general practitioner; UCD, user-centred design

## Additional file

Additional file 1: Chronic care in the Netherlands. (DOCX 31 kb)
