# Supplementary material for: Expectations and needs of patients with a chronic disease toward self-management and eHealth for self-management purposes
Source: BMC Health Serv Res. 2016 Jul 8;16:232. doi: 10.1186/s12913-016-1484-5 (PMC4938915; doi:10.1186/s12913-016-1484-5)
Supplement: Additional file 1: — Chronic care in the Netherlands. (DOCX 31 kb) [file 12913_2016_1484_MOESM1_ESM.docx]

**Supplementary file 1** Chronic care in the Netherlands.

**Chronic care in the Netherlands**

The provision of chronic care in the Netherlands is mainly organized in primary care. Every Dutch citizen is obliged to have a basic health-care insurance. In this basic insurance, general practice consultations are covered. In 2008, a national strategy on chronic disease management was launched by the Ministry of Health, Welfare and Sport. In this strategy, a bundled payment was introduced to facilitate multidisciplinary collaboration in disease management programmes. Moreover, care standards to stipulate the minimum required patient services to be covered and authorized by carers organizations were developed for diabetes, COPD and cardiovascular risk management (CVRM).
